# Unresolved Questions in Rheumatology: Motion for Debate: The Data Support Evidence-Based Management Recommendations for Cardiovascular Disease in Rheumatoid Arthritis

**DOI:** 10.1002/art.37975

**Published:** 2013-07-02

**Authors:** Daniel H Solomon, Mike J L Peters, Michael T Nurmohamed, Will Dixon

**Affiliations:** 1 Brigham and Women’s Hospital and Harvard Medical SchoolBoston, Massachusetts; 2 Jan van Breemen Research Institute | Reade and VU University Medical CentreAmsterdam, The Netherlands; 3 University of ManchesterManchester, UK

## *Presentation of the debate, Dr. Solomon:* Introduction to the motion

Cardiovascular disease (CVD) is a major source of morbidity and mortality in rheumatoid arthritis (RA). While we know much about the epidemiology and biology of this association, less is known about the treatment. This debate focuses on the management of CVD in RA: Do we know enough to set forth evidence-based recommendations? Underpinning this debate are many challenging scientific and clinical issues. First, should management focus on traditional cardiovascular risk factors or on inflammation? Second, should management of traditional cardiovascular risk factors be tailored to patients with RA, modifying treatment thresholds and targets, or are general population recommendations adequate? Third, should RA treatment be tailored to patients at risk of CVD?

While these questions cannot be decided by a debate, our guest debaters have brought evidence to bear on many aspects of these topics. We are sure that you will enjoy the spirited back and forth and will come away with your own opinion of these issues.

## *In support, Drs. Peters and Nurmohamed:* Cardiovascular risk prevention should constitute a key goal of management in RA

Cardiovascular disease is a major source of morbidity and mortality in RA and may equal the (contemporary) CVD burden in diabetes mellitus, a well-established risk factor for CVD (1–4). Currently, cardiovascular risk in diabetes mellitus is substantially lower than during previous decades as a result of effective implementation of strategies to accomplish good glycemic control and, in particular, optimal cardiovascular risk management, with statin treatment and blood pressure reduction being key drivers of this effect (5). In RA, the magnitude of CVD has not appreciably changed over the last decades (6,7). Despite this well-established higher cardiovascular risk, a significant proportion of RA patients still receive no or suboptimal cardiovascular risk management (8–9).

Based on this evidence, we can no longer bury our heads in the sand and pretend that cardiovascular risk management should not be part of our agenda. Cardiovascular risk prevention (i.e., targeting preventive strategies toward high-risk individuals) should constitute a key goal of management in RA. A task force of the European League Against Rheumatism (EULAR) has provided evidence- and expert opinion–based recommendations to help clinicians assess and control cardiovascular risk in RA (11). But, who should be screened, how often, and by whom? Which CVD risk prediction chart should be used? And, what should be the targets or thresholds for treatment with statins and antihypertensive agents?

In RA, well-established cardiovascular risk factors, such as smoking, dyslipidemia, hypertension, diabetes mellitus, and decreased physical activity, occur more frequently (12–14). With information on age, sex, smoking status, lipid levels, and blood pressure, the 10-year absolute risk of a (fatal) cardiovascular event can be calculated with the use of established CVD risk prediction charts (e.g., the Systematic Coronary Risk Evaluation [SCORE] or the Framingham Risk Score) (15,16). Cardiovascular risk management can be easily incorporated into routine visits by adding the measurement of blood pressure and nonfasting total cholesterol and high-density lipoprotein (HDL) cholesterol levels to routine blood testing (17). Of note, recent observations support the use of the total cholesterol–to–HDL cholesterol ratio as the most stable prognostic cardiovascular indicator in RA (18).

All RA patients should receive evidence-based advice with regard to smoking, physical activity, and weight control, and if required based on the absolute cardiovascular risk, adequate management of that risk. Similarly, we underscore the need to assess cardiovascular risk factors in all RA patients, and we encourage clinicians to initiate statin treatment and blood pressure reduction according to national guidelines to reduce the cardiovascular risk. One could argue that we should await intervention trials with statins and/or antihypertensive agents and CVD end points in RA before conclusions about their efficacy can be reached. However, the efficacy of statins and their reduction of clinical end points in RA are probably at least equivalent to their effects in the general population (19,20). Indeed, the effects of statins as well as some antihypertensive agents (i.e., angiotensin-converting enzyme inhibitors and angiotensin blockers) might be more pronounced in RA, since their additional beneficial effects include antiinflammatory properties (21,22). Withholding treatment of proven efficacy (i.e., statins and/or antihypertensive agents) from RA patients, who are already known to be at increased cardiovascular risk, is unethical.

So far, there is no substantial evidence for the use of lower treatment targets for statins and/or antihypertensive agents in RA as compared with those used in the general population. There is no indication for the use of aspirin for primary prevention of CVD in RA.

To ensure sufficient uptake of cardiovascular risk prevention, we have recommended that patients receive a yearly cardiovascular risk assessment (11), but we recognize that in patients with low cardiovascular risk who have low levels of disease activity, a lower frequency of assessment could be adopted.

Traditional cardiovascular risk factors, however, account for only part of the excess cardiovascular risk in RA (22). Inflammation is also important, as it accelerates atherosclerosis, either directly or via effects on cardiovascular risk factors (23). Hence, tight disease control and adequate suppression of the inflammatory process is crucial for lowering CVD risk in RA. Early and effective antirheumatic treatment has been shown to be associated with a lower cardiovascular risk, with methotrexate and tumor necrosis factor α (TNFα)–blocking therapy having the best available data, accepting that even here, we lack clinical trials showing definitive proof (24,25). Interestingly, it is possible that cardiovascular risk is reduced only in patients who respond to TNFα-blocking therapy (26).

One could argue that the introduction of the current powerful biologic therapies will neutralize the excess cardiovascular risk in RA, making cardiovascular risk management redundant. Data to support this notion are lacking, however, and recent risk estimates still demonstrate an ∼50% increased risk of CVD. Moreover, in sharp contrast to the much cheaper and more widely tested generic lipid- and blood pressure–lowering agents (e.g., in Europe, simvastatin costs at little as €5 per year, compared to more than €10,000 per year for TNFα-blocking agents), these new agents are very expensive and are not effective in ∼30% of RA patients (27). In addition, despite the ability to strongly suppress disease activity, systemic levels of cytokines sometimes remain high relative to those in non-RA patients. Hence, even RA patients receiving such treatment may still be at elevated risk of CVD.

Nonsteroidal antiinflammatory drugs (NSAIDs), coxibs, and corticosteroids may influence cardiovascular risk in two competing ways. NSAIDs and coxibs exert prothrombotic effects, while corticosteroids have deleterious effects on lipids, insulin resistance, blood pressure, and obesity (28,29). In contrast, these drugs may actually reduce cardiovascular risk by suppressing inflammation, which may improve mobility. Yet, conclusive evidence is not available. With these agents, we recommend the use of the lowest dose possible for the shortest period possible.

Given the complex interplay between disease activity, cardiovascular risk, and the effect of antirheumatic treatment, rheumatologists are well positioned to perform cardiovascular risk assessments, considering that blood samples need to be obtained anyway and blood pressure measurement is noninvasive. Alternatively, cardiovascular risk management could be done in coordination with primary care physicians, who are experts in this process.

Which risk scoring models should be used? We acknowledge that a CVD risk prediction chart is lacking for RA and that calibration of existing risk prediction charts for RA has not been done. There are several options for dealing with this. First, we can use existing CVD risk prediction charts until a model has been developed that is appropriate for RA. This will take many years, however, and in the meantime, many patients will receive suboptimal cardiovascular risk management because existing CVD risk prediction charts underestimate the true cardiovascular risk in RA (30). Second, we can use lower treatment targets for all RA patients, but evidence to support this notion is lacking. Moreover, the use of lower treatment targets is expensive and will increase the likelihood of patients’ being harmed by the therapy. Third, the existing risk prediction charts are adapted in such a way that patients with an increased cardiovascular risk receive management.

The EULAR task force thought this last option was the most appropriate and therefore recommended a multiplication factor. The task force selected 3 prognostic CVD markers to identify patients at highest risk of developing CVD: disease duration >10 years, presence of rheumatoid factor (RF) and/or anti–cyclic citrullinated peptide antibodies, and presence of severe disease (e.g., extraarticular manifestations). When patients meet at least 2 of these criteria, the risk estimate calculated with an existing CVD risk prediction chart should be multiplied by a factor of 1.5. For example, consider a 60-year-old female patient with RA who smokes and has a total cholesterol level of 6.8 mmoles/liter, an HDL cholesterol level of 1.1 mmoles/liter, and a systolic blood pressure of 170 mm Hg. According to the SCORE model (https://escol.escardio.org/Heartscore/), this patient has an 8% risk of experiencing a fatal cardiovascular event within 10 years. If this patient is IgM-RF positive and has an RA duration of >10 years, we need to multiply this risk by 1.5, which results in a 12% chance of a fatal cardiovascular event within 10 years. According to several European guidelines, treatment with statins and/or antihypertensive agents will be started when the CVD risk score is >10%, provided that the systolic blood pressure is >140 mm Hg and/or the low-density lipoprotein (LDL) cholesterol level is >2.5 mmoles/liter (96.7 mg/dl).

We acknowledge that the multiplication factor of 1.5, which was derived from relevant standardized mortality ratios, is a matter of debate, since there is a paucity of prospective cohorts to enable the more usual approach to defining multiplication factors. A recent study of general practitioners in the UK provided additional support for a multiplication factor of ∼1.5 (31). Finally, Crowson and colleagues (30) recently demonstrated that cardiovascular risk appears to be most pronounced among RA patients who are RF positive, older, and have severe disease. Their findings are consistent with those upon which the EULAR recommendations were based.

In summary, the increased CVD risk in RA is indisputable and is due to both an excess of “traditional” cardiovascular risk factors and the underlying chronic inflammatory process. We acknowledge the need for CVD prevention trials and RA-specific CVD risk prediction charts, but we cannot wait the many years it would take before these are available. We should incorporate cardiovascular risk management now—thereby reducing the CVD risk burden in our patients—and continue to evaluate its effectiveness over time.

## *In opposition, Dr. Dixon:* Cardiovascular risk intervention requires a balance of benefits, harms, and cost-effectiveness

Evidence-based medicine has been defined as “the conscientious, explicit, and judicious use of current best evidence in making decisions about the care of individual patients” (1). This definition incorporates the use of clinical expertise and external evidence, in particular from patient-centered clinical research, in order to come to optimal decisions about interventions for our patients (1). Where there is a burden of disease, we need to know with confidence how best to improve health.

It has been established that in patients with RA, CVD rates are increased as compared to those in the general population. This is manifested as an increased incidence of myocardial infarction and stroke (2), and increased rates of death from cardiovascular causes (3). Guidelines assimilating and interpreting the expanding data are welcome: ∼15 new articles have been published on this topic every month over the last 5 years (4). The current EULAR guidelines (5), which were published 2 years ago, have already been cited over 200 times, reflecting the usefulness of synthesizing the evidence into guideline form. Critically appraising the evidence from all available sources is challenging, but vital, if we are to understand whether we have a proven, effective intervention for reducing cardiovascular risk that is worth implementing in daily practice.

It can be helpful to separate the debate title into a series of sequential questions. First, is cardiovascular disease increased? (Answer: Yes—see above.) Second, by what mechanism is CVD increased? Third, what is the evidence that intervention lowers the risk of CVD, and to what extent is it lowered? And last, is that intervention worthwhile? Typically, the evidence base weakens as we move through this series of questions. Each of these questions will be explored in turn.

Cardiovascular risk in patients with RA is increased by a variety of mechanisms, including both traditional (smoking, hypertension, dyslipidemia) and nontraditional (inflammation, medication) risk factors. This topic has been reviewed extensively elsewhere (6,7). Interventions can thus be targeted to many areas of potentially increased risk. However, the multiple routes from RA to increased CVD risk means that it cannot be assumed that intervening in one pathway has a predictable clinical outcome. There are many examples where pharmacologic interventions have had unpredictable, bidirectional, or even paradoxical effects, for example, increased suicidality with the use of antidepressants, atypical femoral fractures with bisphosphonates, and new-onset psoriaform lesions with anti-TNF therapy (8). The debate must therefore focus on whether we have sufficient evidence that an intervention targeted at one of the many risk pathways truly reduces the burden of CVD. Surrogate end points are useful, but the clinical end points are the ones that matter.

What, then, is the evidence that intervention lowers the incidence of CVD and to what extent is it lowered? This can be considered with respect to interventions targeted at traditional cardiovascular risk factors and nontraditional risk factors. At present, there are no published interventional studies (randomized controlled trials [RCTs]) examining the efficacy of statins or antihypertensive agents on clinical cardiovascular end points exclusively in RA populations. Similarly, there are no studies of weight reduction or smoking cessation examining such end points in RA. There are therefore no data from RCTs in this particular disease population to support the use of these primary prevention measures. This does not mean that we have no information, as we can extrapolate the results from other study designs and other populations.

Observational studies of smoking cessation in patients with coronary heart disease report a 36% reduction in the relative risk of death in those who quit as compared to those who continue to smoke (9). All rheumatologists will accept guidelines that advise smoking cessation in patients with RA, even though the research was conducted in the general population. RCTs of statins and antihypertensive agents have shown a clear reduction of cardiovascular end points in populations with existing CVD (secondary prevention). Evidence of a positive impact weakens among populations with no prevalent CVD (primary prevention). Statins have been shown to have a positive effect in high-risk patients without prevalent CVD, but caution is advised in patients with low cardiovascular risk (10). Primary prevention of CVD in the low-risk general population is not currently advocated because the background rate, and thus, the absolute risk reduction, of cardiovascular disease is low. Many patients would need to be treated—with the associated side effects and cost implications—in order to avoid 1 case of CVD.

The difficult balance in RA rests here, among patients with a lower baseline risk of CVD. Following the evidence cited above, namely, that RA is estimated to confer an additional 50% risk of CVD beyond that conferred by the traditional risk factors, the EULAR guidelines advocate multiplying an individual’s cardiovascular risk prediction score by 1.5 (5).This additional risk can move an individual across the risk, and thus the treatment, threshold. The example given in the recommendations describes a female patient with traditional risk factors that would confer a 7% risk of a fatal cardiovascular event within 10 years in the general population. That probability is then multiplied by 1.5 because of her RA, giving her a resultant 10-year risk of experiencing a fatal cardiovascular event of 10.5%. This moves her into a risk category that requires intervention.

Importantly, the advocated treatment at this point is targeted to the traditional risk factors. But, is this increased risk conferred by RA modifiable by interventions targeted to lipids or blood pressure? Might statins work less well in RA patients than in the general population because, say, inflammation drives CVD progression in RA more than lipid metabolism does? If statins do have a positive effect, is this benefit seen at a particular stage of disease? Might it be that, once the increased CVD is established in RA, reducing lipid levels can slow, but cannot reverse, the process? These questions remain unanswered and frame the research agenda in this area.

Another option for reducing CVD in patients with RA is to treat the nontraditional risk factors, such as inflammation. There is evidence to support this. All studies, bar one, in a recent meta-analysis showed a reduced risk of cardiovascular events in patients treated with methotrexate (overall relative risk 0.79 [95% confidence interval 0.73–0.87]) (11). The magnitude of the reduced risk is clinically meaningful. Although bias is possible (such as residual confounding from steroid use), a 20% cardiovascular risk reduction would be a welcome consequence of treating the joint symptoms. Recommendations cite this drug as having the best evidence for risk reduction. While this may be true, we must be cautious not to infer that methotrexate leads to a greater risk reduction than sulfasalazine or leflunomide does. There have been few studies examining the cardiovascular effects of these treatments, and no comparative studies. If we are to choose the optimal treatment that reduces disease severity and cardiovascular risk, we need to acquire this information.

Predicting the cardiovascular effects of anti-TNF therapy is difficult because TNF is involved at many stages of the pathophysiologic pathway (12). It may even have bidirectional effects, for example, by reducing inflammation but increasing lipid levels. Interestingly, observational studies of cardiovascular outcomes following anti-TNF therapy have led to discrepant, apparently conflicting, results (for tabulation, see ref.^13^). Reported associations range from a 4–5-fold lower rate (14,15) to a 70% increased rate of incident cardiovascular events (16). One reason for the discrepancy becomes clear as these studies are compared. Although all studies address the influence of anti-TNF therapy on CVD in patients with RA, no two studies have the same comparator group, the same definition of cardiovascular outcome, and adjust for the same confounders. Furthermore, there are additional differences between studies in terms of the penetration of drug use, the average disease duration, and more. At present, we still do not know the effects of anti-TNF therapy on cardiovascular outcomes. Consensus in this area might be possible, but investigators need to work together to address comparable research questions (17).

The last and most important question is whether any intervention is worthwhile—ultimately, it is a balance of benefits, harms, and cost-effectiveness. This is not always an easy balance, particularly since each outcome is measured in different units and information can be missing. Nevertheless, despite evidence gaps for a range of questions in RA populations, we can still be proactive in reducing cardiovascular risk. Lifestyle changes, such as weight reduction and smoking cessation, offer a wealth of positive benefits, and so we must continue (or start!) to provide such advice. Tight control of disease activity has already been established as the standard of care for patients with RA in order to optimize the reduction of disease progression in their joints. Cardiovascular benefits can be readily accepted as an added benefit. However, intervening to treat traditional risk factors in patients with RA at a stage at which intervention would not be advocated in the general population does not yet have a supporting evidence base. Before committing large numbers of RA patients to such interventions, we need to have quantified the benefits and harms.

## *In support, Dr. Peters and Nurmohamed:* Rebuttal

Dr. Dixon begins his argument confirming that RA patients are at increased cardiovascular risk. However, we must ask ourselves whether physicians are aware of the increased cardiovascular risk conferred by RA. A recent study suggests that there is poor cardiovascular risk management by primary care physicians as well as rheumatologists. This clearly demonstrates the need for better education to improve awareness of cardiovascular risk in RA (32).

His argument continues by highlighting the fact that cardiovascular risk in RA is attributed not only to classic risk factors, but also to nonclassic risk factors, including systemic inflammation and antirheumatic treatment. The latter point about medications may complicate our understanding of the role of RA-specific treatment in cardiovascular risk in RA. While we agree to some extent, this argument applies to almost all diseases and therapeutic interventions. But, should we, because of our lack of knowledge about its true effect on the CVD burden in RA, avoid statin therapy in RA patients at high risk of CVD? We respectfully disagree.

Dr. Dixon is right, in that the extrapolation of intervention effects from non-RA patients to those with RA is not supported by the findings of RCTs. However, the same argument can be made about the use of many treatments in groups that have been underrepresented in trials, such as women and many ethnic minorities. We should not withhold treatment of cardiovascular risk factors from these patients if knowledge of the pathobiology of CVD supports extrapolation of the findings of large intervention trials. Such extrapolations are integral to the practice of medicine, and the type of rationale to support them is part of evidence-based medicine (33). The only patient category at this time where extrapolations of large cardiovascular intervention trials have failed are those with end-stage renal disease, who seem to be “beyond repair” in terms of cardiovascular damage. There is no rationale for why chronic inflammation or other RA-related factors would render statins or antihypertensive agents ineffective. This does not imply that trials should not be performed, but it does mean that RA patients should be given the benefit of the doubt.

In addition, Dr. Dixon adds examples from several pharmacologic interventions in which there were potential adverse effects, for example, increased suicidality with antidepressant therapy. Does this mean that we should not treat patients who are depressed? Similarly, should we avoid statin therapy in RA patients at high risk of CVD because of its possible myopathy? We disagree with this approach. Lack of data from RCTs on the effects and/or side effects of statin therapy or other cardiopreventive drugs in RA should not deter us from trying to bring down the 50% higher risk of CVD in patients with RA. Additionally, there are no data that would lead us to believe that statin therapy is less effective in RA, and the potential hazards of statin therapy seem to be extremely small in relation to the clear benefits in many circumstances (19,34). We concur that specific trial data conducted in RA patients will improve the evidence base on this clinically relevant topic. Until such trials have been performed, however, withholding cardiopreventive drugs that are very likely to work in the RA population simply seems unethical.

Dr. Dixon’s debate position focuses on the lack of evidence to support treatment of classic risk factors in patients with RA at a stage where intervention would not be advocated in the general population. Here, we would like to emphasize that cardiovascular risk management in RA should be done according to national guidelines, no different from that in the general population; that is, only when the global 10-year risk of a fatal cardiovascular event is ≥10% is treatment instituted at the point when the blood pressure and/or lipid levels are increased. Importantly, recently published data underscore the fact that well-established CVD risk prediction charts, such as the Framingham Risk Score and the Reynolds Risk Score, underestimate the true cardiovascular risk in patients with RA, indicating that the (conservative) 1.5 multiplier is needed in order to make an appropriate estimation of the true cardiovascular risk in RA (30). To deal with this and because accurate assessment of cardiovascular risk depends on the characteristics of the RA, the EULAR task force favored individualized risk assessment.

In conclusion, whichever approach is taken, the recognition of RA as a cardiovascular risk factor is important. Availability of simple CVD risk prediction charts will lead to wider implementation of risk-modification strategies in routine clinical care. The extrapolation of the favorable effects of statins and antihypertensive agents to patients with RA is rational and reasonable and is in perfect agreement with what David Sackett described as an appropriate and comprehensive definition of evidence-based medicine in the very same paper Dr. Dixon uses as his first reference (33).

## *In opposition, Dr. Dixon:* Rebuttal

There is significant overlap in the pro and con positions in the first half of the debate. Readers might even be forgiven for missing the area of contention altogether. To illustrate, I agree with most of the concluding paragraph of Drs. Peters and Nurmohamed’s argument. We agree that (a) there is an increased risk of CVD in patients with RA, (b) this risk is due to both an excess of “traditional” cardiovascular risk factors and the underlying chronic inflammatory process, and (c) while there is a need for CVD prevention trials in RA as well as RA-specific CVD risk prediction charts, we must optimize the treatment we prescribe now. Where we disagree is over which cardiovascular risk management interventions should be incorporated, in whom, and importantly, the focus of this debate, on what evidence?

Let me first underline our agreement concerning cardiovascular risk management. Patients with RA who fulfill general population criteria for cardiovascular risk reduction should receive interventions (smoking cessation, weight reduction, blood pressure control, lipid-lowering therapy), irrespective of their RA. Patients with RA and obesity should be given advice about weight management. While their arthritis might make exercise more difficult, weight reduction should remain a goal. Smokers should be encouraged to stop smoking, regardless of whether they have RA. Hypertension or dyslipidemia should also be treated, if appropriate, based on absolute cardiovascular risk, using validated prediction models such as the Framingham Risk Score. We all agree that the absence of evidence for the reduction of cardiovascular end points in the RA population should not stop us from treating cardiovascular risk factors as we would in the general population. We also agree that we should treat active inflammation, which will not only benefit the joint disease, but has also been proven to have a beneficial effect on cardiovascular end points. Which treatment has the most benefit for this important comorbid condition remains to be seen and should be carefully investigated.

The critical gap in our knowledge is whether we should treat cardiovascular risk factors in patients who would not previously have fulfilled the criteria. Applying a multiplication factor of 1.5 to a risk score of 8% would tip some people beyond the treatment threshold, but is this multiplication factor appropriate? Does RA generate its excess cardiovascular risk only by multiplying the risk conferred by the traditional cardiovascular risk factors, say cholesterol, as the algorithm suggests? If so, targeting the cholesterol would be appropriate and would remove the cardiovascular risk of RA. This multiplicative model assumes that RA acts solely via the pathway of traditional risk factors through an augmented effect of these established risks. We have already all agreed that this is not the case.

An alternative to the multiplicative model is that RA is an additive risk factor. In other words, a raised cholesterol level causes *x* additional heart attacks per 1,000 patients, and the presence of RA would add a further *y* cases. Treating the raised cholesterol level in this instance would have the same impact as it does in the general population. Because such a treatment was deemed inappropriate in large general population studies, it would be no more appropriate in patients with RA, since the benefits, harms, and costs are unchanged. The truth is likely to fall between these models, leaving uncertainty about appropriate treatment in this group.

This is the difficult area where evidence is lacking, to reiterate: patients who would not meet traditional treatment thresholds for cardiovascular preventive treatments, but who, by nature of their RA, are accepted to be at higher risk. We need to know how best to intervene to minimize their cardiovascular risk. But how can this be determined? An RCT examining primary prevention in RA using, say, statins and powered to detect cardiovascular end points would allow us to answer this question. Indeed, the Trial of Atorvastatin for the Primary Prevention of Cardiovascular Events in Patients with Rheumatoid Arthritis (TRACE-RA) was established in the UK to address just this question. However, despite being the largest academically led RCT of RA in the UK, the study was terminated early, after more than 3,000 patients of the 4,000 target population had been recruited, due to a low incidence of the primary cardiovascular end point. Projected figures showed insufficient power to reach a clear conclusion (Symmons D, Kitas G, Belch J: personal communication). Despite its early discontinuation, the fact that TRACE-RA patients (irrespective of treatment) had a low absolute risk of cardiovascular end points is encouraging. Decisions about the value of interventions are based on absolute risk reduction or the number needed to treat. The low overall incidence means that any difference between the arms is less likely to be clinically meaningful.

If RCTs are impractical, how do we fill this gap in knowledge? Observational research can measure the impact of statins in patients with RA, but interpretation of any results suffers from confounding by indication. The treatment group is given statins for a particular reason (dyslipidemia and or known CVD) and is thus expected to have a higher risk of CVD compared to the comparator group (no statins), who does not have dyslipidemia or known CVD. One possible solution is to run pragmatic trials within observational settings, randomizing patients to treatment, then collecting information on outcomes as part of routine clinical practice (18). This will take a concerted effort across the rheumatology community plus development of infrastructure, but it is a goal worth pursuing.

This debate was framed around the evidence to support treatment recommendations. Despite some discussion about the multiplication factor for risk prediction and the treatment threshold, we agree on most things. The 12–25% of patients with RA who currently smoke (19) need to be encouraged to stop. Over half of patients with RA have a body mass index of >25 kg/m^2^, and 70% perform no regular physical activity (20). Healthy lifestyles leading to a desirable body weight, a healthy diet, regular exercise, and not smoking could account for an 84% reduction in cardiovascular risk, and yet, few people fall into this category (21). Focused management of cardiovascular risk factors that warrant intervention in the general population can make a big difference in RA. Meanwhile, researchers should continue to explore the comparative effectiveness of treatment with disease-modifying antirheumatic drugs for cardiovascular end points and evaluate the balance of benefits and harms for primary prevention in RA.

## AUTHOR CONTRIBUTIONS

All authors drafted their individual section of the article, revised it critically for important intellectual content, and approved the final version to be published.
